# La veine cave inférieure gauche et la greffe rénale

**DOI:** 10.11604/pamj.2019.34.109.20643

**Published:** 2019-10-24

**Authors:** Khalid Elmortaji, Adil Debbagh, Mohamed Dakir, Rachid Aboutaieb, Fethi Meziane

**Affiliations:** 1 Service d’Urologie, Chu Ibn Rochd, Casablanca, Maroc

**Keywords:** Transplantation rénale, transposition gauche, veine cave inférieure, Kidney transplantation, left sided, left inferior vena cava

## Abstract

Les anomalies congénitales des gros vaisseaux sont rares, dont la veine cave inférieure gauche, occupe le 2^ème^ rang après la duplicité. Elles représentent un défi dans certaines chirurgies urologiques et vasculaires. Nous rapportons le cas d'un donneur vivant pour greffe rénale avec découverte d'une Veine Cave Inférieure (VCI) gauche lors du bilan radiologique pré-greffe.

## Abstract (en)

Congenital anomalies of the large vessels are rare. Left inferior vena cava is the second most common anomaly after duplication. They are a challenge that some Departments of Urology and Vascular Disease face. We report the case of a patient admitted for kidney transplantation in whom left inferior vena cava was detected on radiological evaluation performed in the preoperative period.

## Introduction

La transposition de la veine cave inférieure (VCI) est une situation exceptionnelle occupant le 2^ème^ rang des malformations de la VCI; souvent de découverte fortuite lors d’un bilan radiologique, la connaissance de ce type de variante anatomique est indispensable pour tout chirurgien urologue et vasculaire. Nous rapportons le cas d’un donneur vivant pour greffe rénale avec découverte d’une VCI gauche lors du bilan radiologique pré-greffe.

## Patient et observation

**Figure 1:**
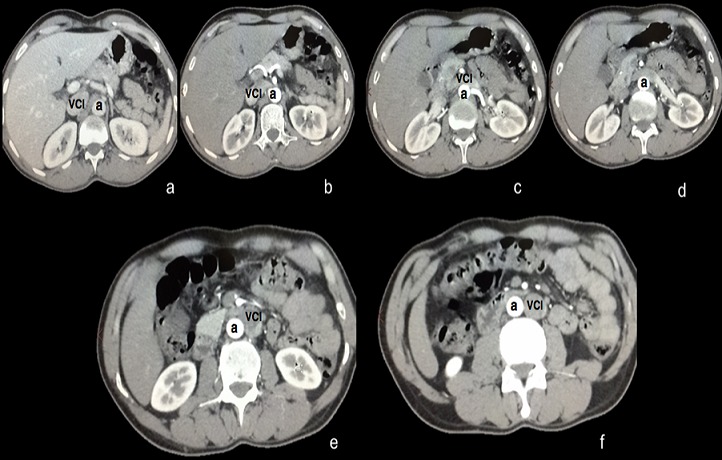
Coupe scanographique transversale montrant le trajet de la veine cave inférieure; a: Aorte abdominale, VCI: Veine Cave Inférieure

**Figure 2:**
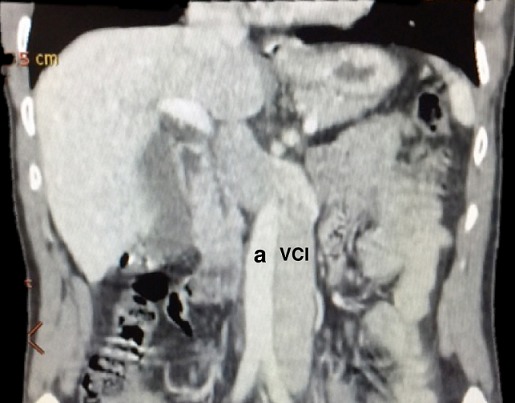
Coupe scanographique coronale passant par l’axe vasculaire

Il s’agit d’un homme âgé de 50 ans, sans antécédents particuliers, admis pour don de rein en vue d’une transplantation rénale. L’examen clinique était sans particularité. Le bilan radiologique pré-greffe fait d’une tomodensitométrie (TDM) abdominale a objectivé une VCI gauche dans sa portion sous rénale (Figure 1) qui se termine à la hauteur de la veine rénale gauche, et se draine en formant un tronc commun avec celle-ci vers la portion pré-hépatique normale de la VCI située à droite, en croisant l’aorte abdominale en avant (Figure 2). Le bilan biologique était normal. Devant la bonne qualité du rein droit par rapport au gauche, et la présence d’une artère et veine uniques de longueur normale, l’indication du prélèvement rénal gauche a été retenue. L’exploration chirurgicale a trouvé une VCI gauche sous rénale sans autres anomalies. Les suites post-opératoires étaient simples chez le donneur, avec une sortie à j3 post-opératoire.

## Discussion

La VCI gauche est une variante anatomique rare avec une incidence de 0,2 - 0,5 % [1]. Pendant la période de l´embryogenèse précoce, le drainage veineux des côtés gauche et droit du corps se fait indépendamment l’un de l’autre. Après la régression de la majorité des veines supracardinales gauches et des veines de l´interconnexion entre les veines sacrocardinales, l’ensemble du drainage veineux du membre inférieur gauche se fait vers le côté droit, en formant ainsi la VCI. Une perturbation lors de ce processus de développement veineux peut entraîner des variantes anatomiques de position, telle que la VCI gauche [1]. Son diagnostic clinique est resté asymptomatique [1]. La TDM constitue l’examen de choix pour détecter ces variations vasculaires avec une sensibilité et une spécificité pour les anomalies artérielles de 91,6 et 98,2% et veineuses de 96,7 et 90% respectivement [2], avec quelques images pièges telle qu’une dilatation urétérale gauche ou une adénopathie, où un œil non expérimenté et non averti peut passer à côté d’une VCI gauche [2]. Chez notre patient et dans la majorité des cas, la VCI gauche croise la face antérieure de l’aorte abdominale à hauteur de la veine rénale gauche pour former la portion normale de la VCI sus rénale [3]. La longueur moyenne de la veine rénale chez les patients avec une VCI gauche est de 1,5cm, ce qui la rend plus courte comparativement à l’état normal [3]. Dans notre cas, la veine rénale gauche avait une longueur de 2cm.

## Conclusion

La présence d’une VCI gauche ne contre-indique pas le prélèvement de rein; cependant la connaissance de ce type de variante reste primordiale imposant une bonne analyse radiologique afin d’éviter d’éventuelles surprises peropératoires.

## Conflits d’intérêts

Les auteurs ne déclarent aucun conflit d'intérêts.
